# Supplementary figures and images for: Adenovirus phagocytosis by neutrophils triggers a pro-inflammatory response
Source: PLoS Pathog. 2026 Apr 6;22(4):e1013504. doi: 10.1371/journal.ppat.1013504 (PMC13102305; doi:10.1371/journal.ppat.1013504)

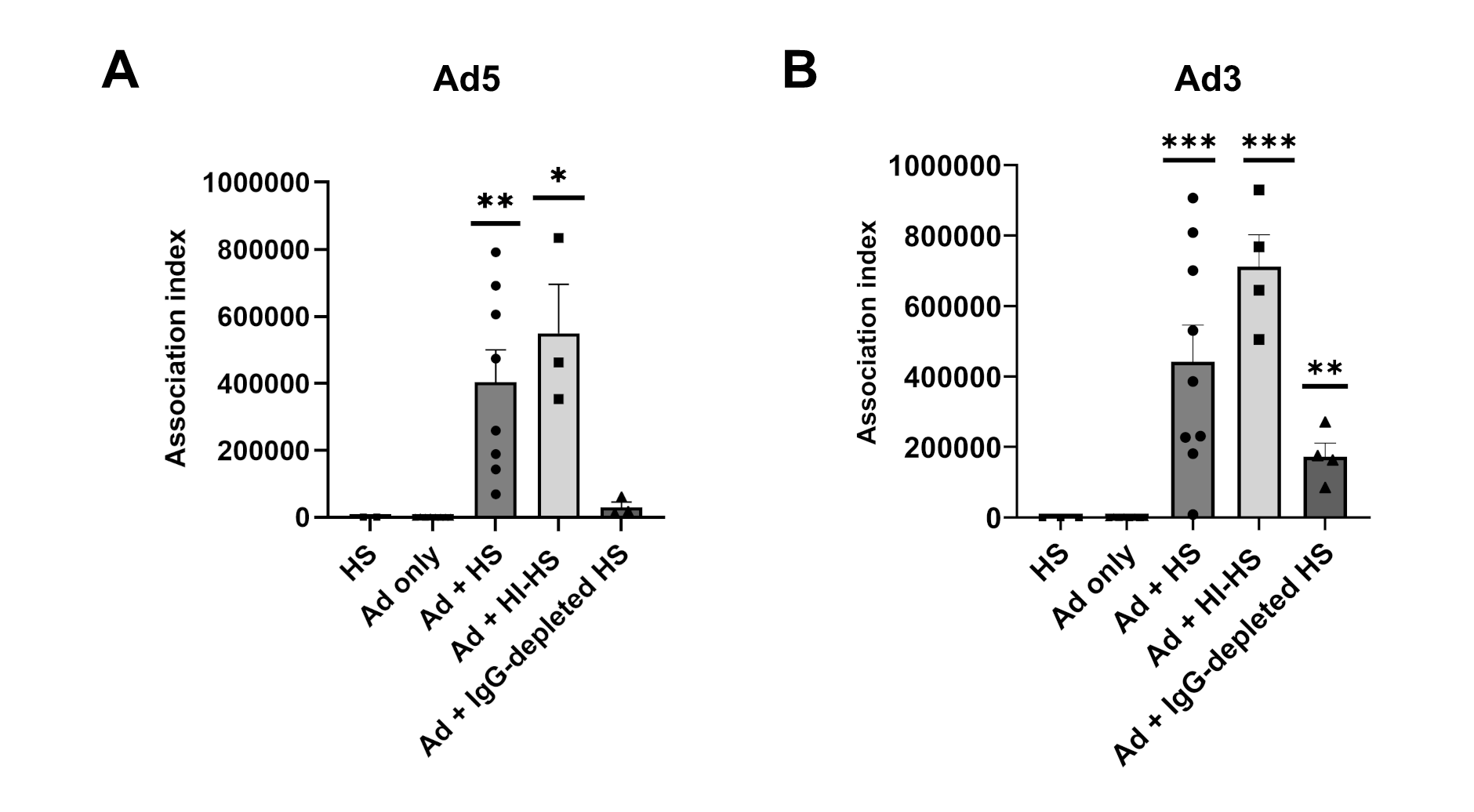

Supplement: S1 Fig — (A) Alexa-488 labeled Ad5 (A) or Ad3 (B) were incubated at 37°C with medium, human serum (HS), heat-inactivated (HI-HS) or IgG-depleted HS. The PLB-985 cells were then exposed to Ads at 4°C to allow cell binding (MOI 104 vp/cell). The results are presented as an association index + SEM (i.e., mean fluorescence intensity multiplied by percentage of positive cells). Each dot represents the association index for one experiment. A Kruskal-Wallis test was performed followed by Dunn’s multiple comparison tests. to compare binding in the HI-HS and IgG-depleted HS conditions relative to the HS condition. *, p < 0.05; **, p < 0.01; ***, p < 0.001. (TIF) [file ppat.1013504.s001.tif]

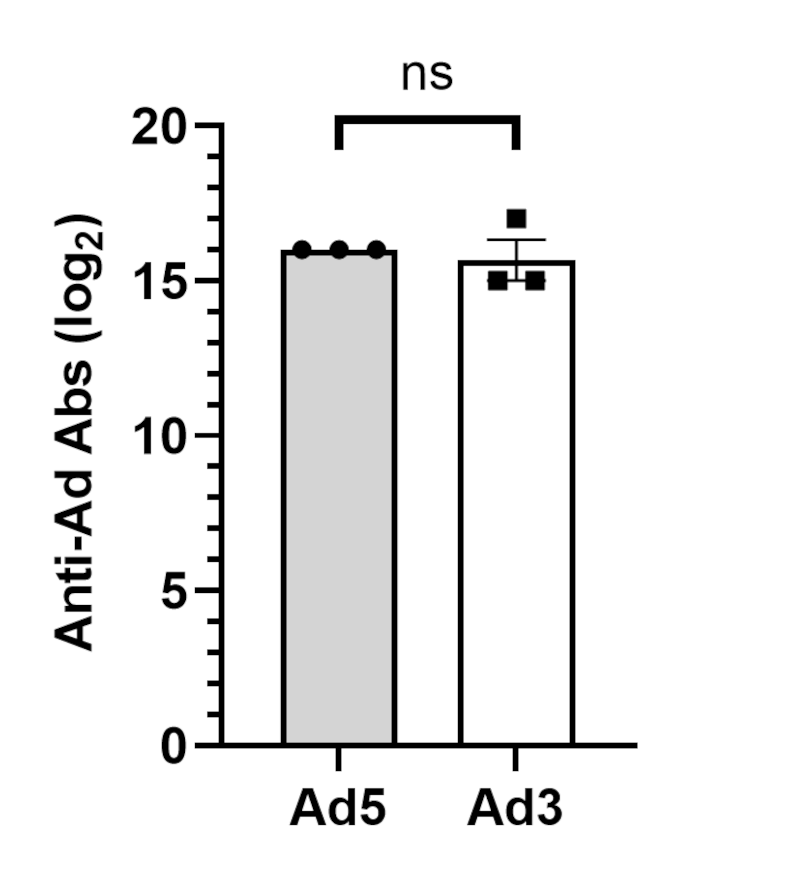

Supplement: S2 Fig — Titers of anti-Ad IgGs in the human serum used for opsonization were determined by ELISA. A Mann-Whitney test showed that there was no significant difference (ns) between the Ad5 and Ad3 titers, expressed as the mean ± SEM on a log2 scale. Three independent experiments were performed. (TIF) [file ppat.1013504.s002.tif]

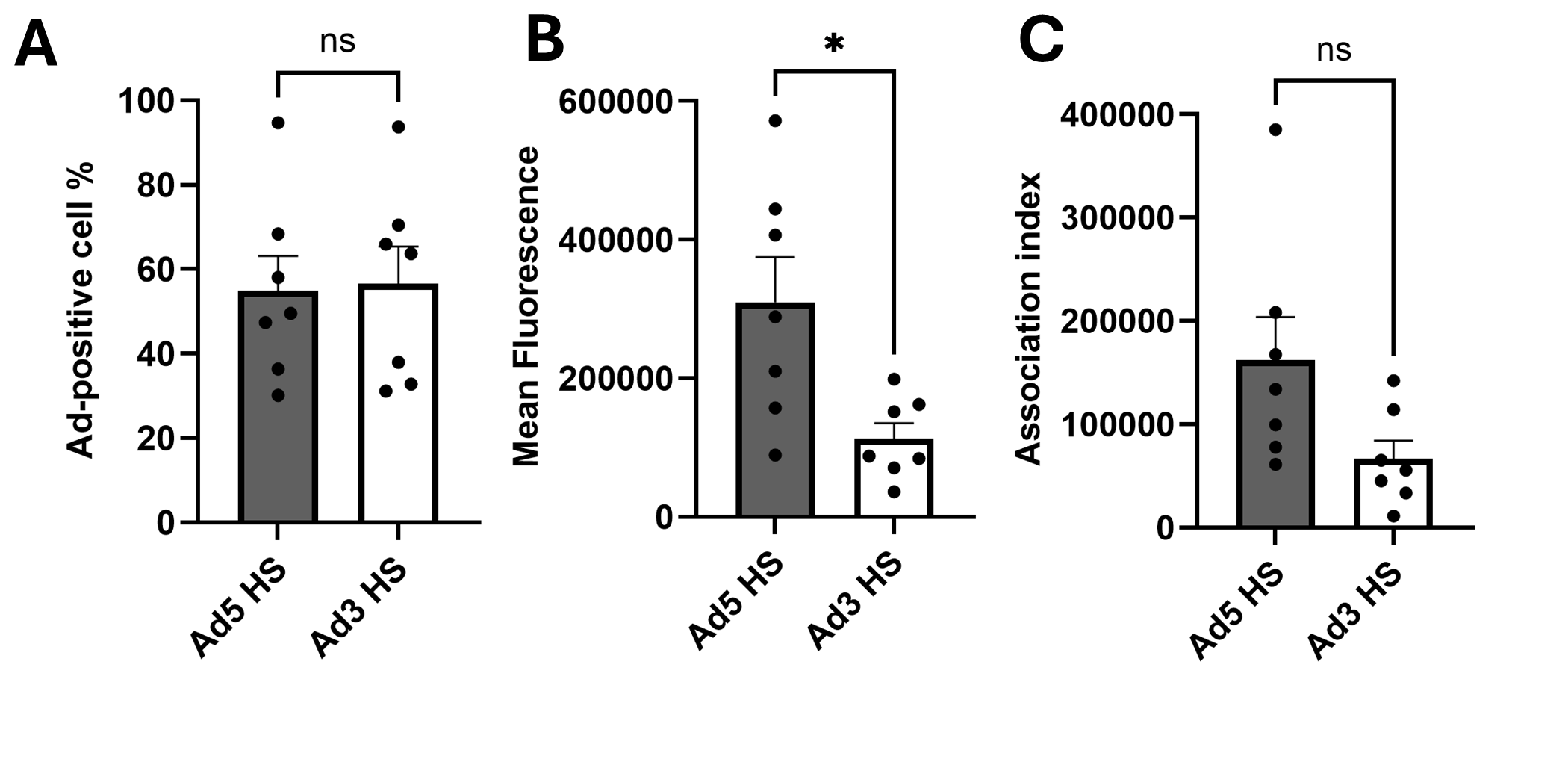

Supplement: S3 Fig — A-488 labeled Ad5 or Ad3 were incubated at 37°C with human serum (HS). PMNs were then exposed to these Ads at 4°C to allow cell binding (MOI 104 vp/cell). (A) Percentage of Ad-positive cells + SEM, indicating the number of cells with Ads at their surface; (B) Mean fluorescence representing the amount of Ad bound per cell; (C) Association index calculated from the previous data. Welch’s t test was performed. ns, non-significant; *, p < 0.05. Seven independent experiments were performed. (TIF) [file ppat.1013504.s003.tif]

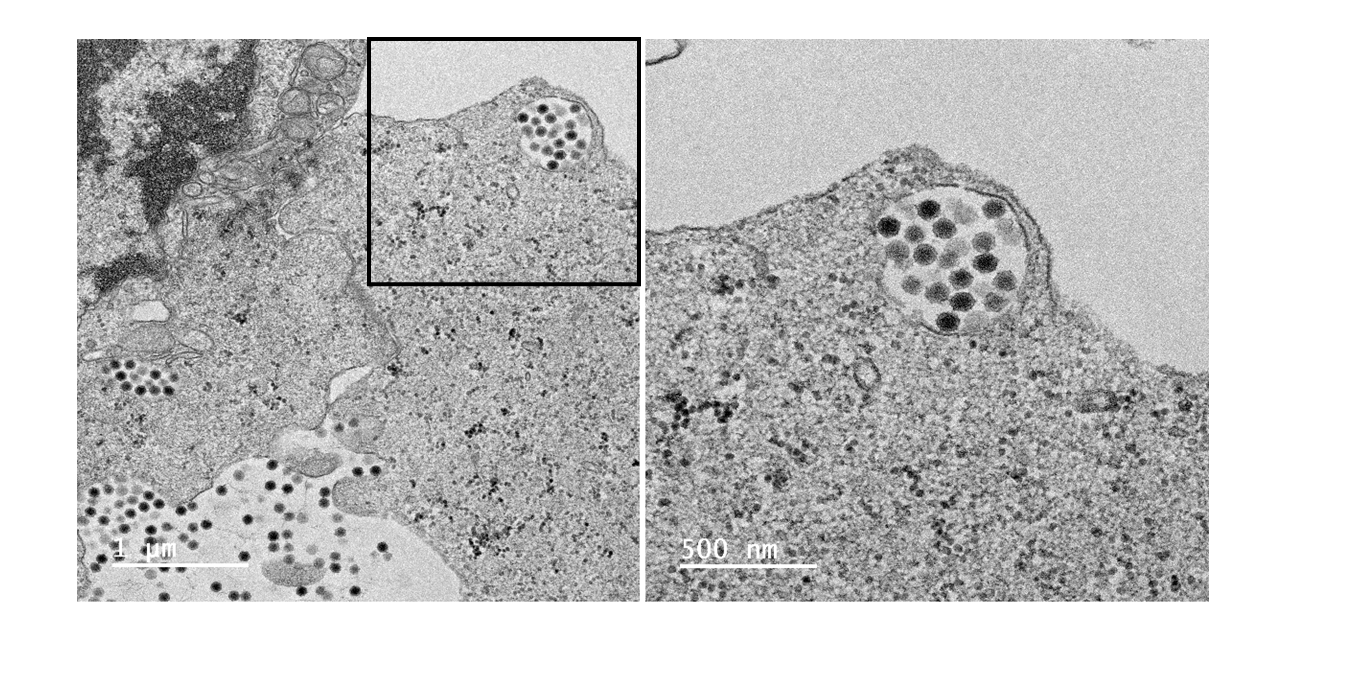

Supplement: S4 Fig — PLB-985 cells were incubated with human serum-opsonized A-488 Ad5 (MOI 104 vp/cell) for 1h at 37°C, then prepared for electronic microscopy. The images show an Ad5-infected cell with a phagosome engulfed viral particles (inset). (TIF) [file ppat.1013504.s004.tif]

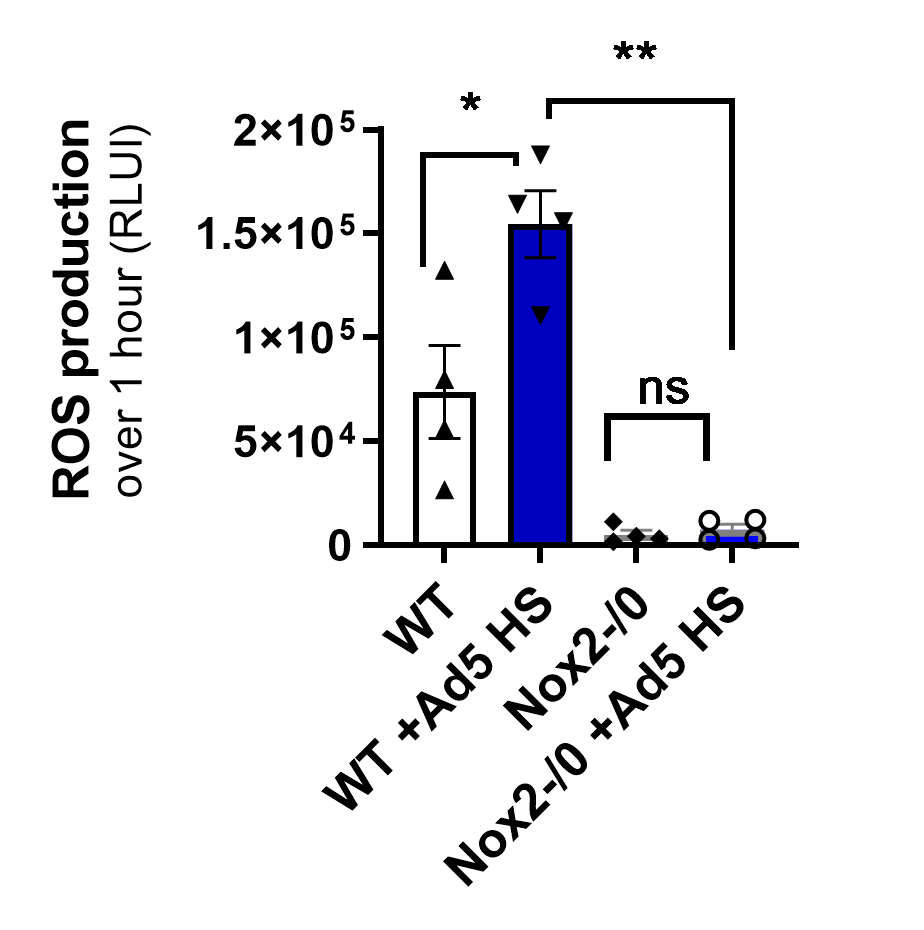

Supplement: S5 Fig — WT & NOX2-/0 PLB-985 cells were incubated with buffer or human serum-opsonized Ad5 (MOI 104 vp/cell). Analysis of ROS production over 1 hour, detected by a luminometry-based test, showed that in WT cells, Ad5 infection resulted in significantly increased ROS production but that in NOX2-deficient PLB-985 cells ROS production did not increase following infection, and indeed remained very low. An Anova-test followed by t-tests with Welch’s correction was performed: ns, non significant; *, p < 0.05; **, p < 0.01. Four independent experiments were conducted with technical duplicates for each condition in every experiment. (TIF) [file ppat.1013504.s005.tif]

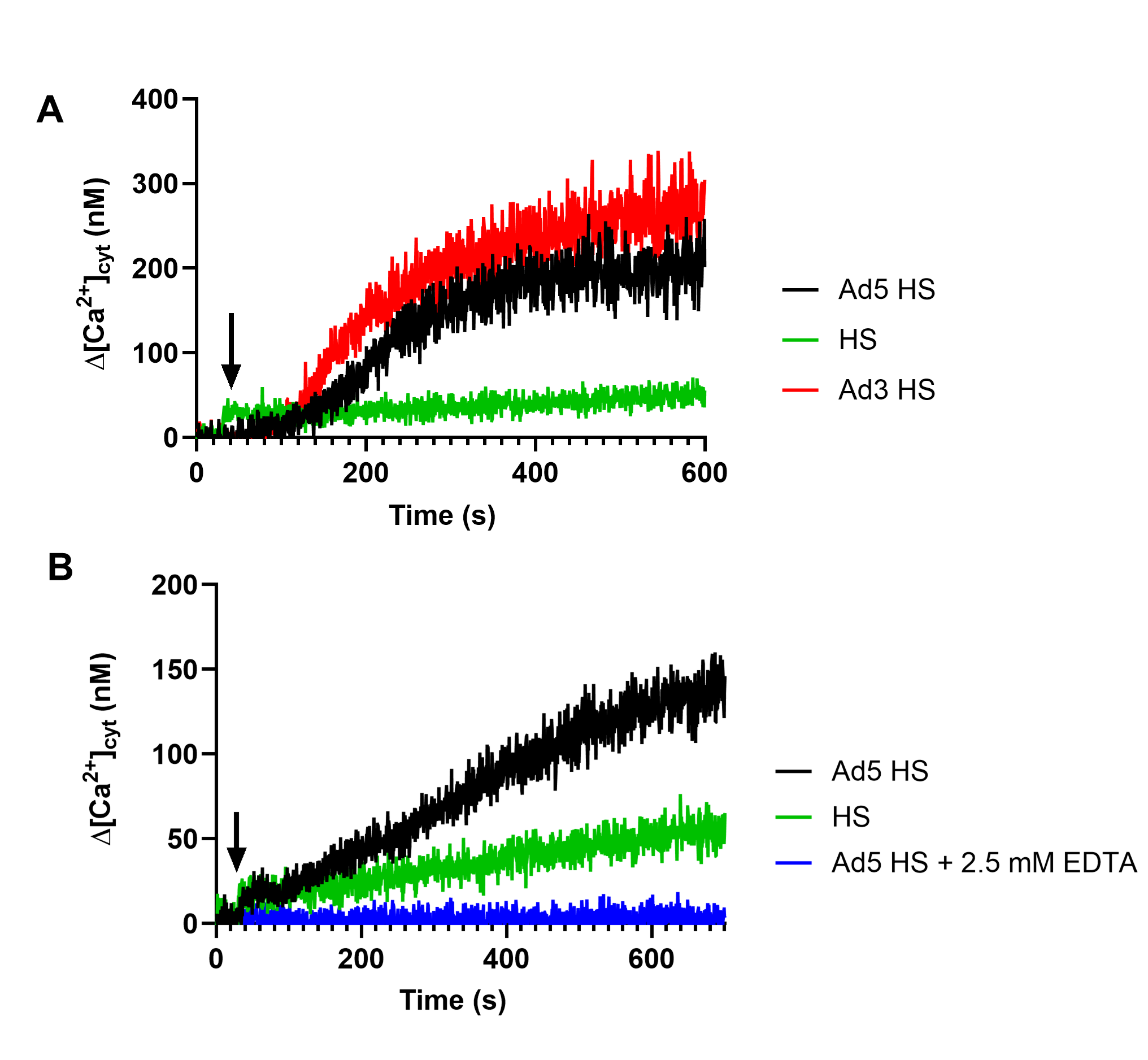

Supplement: S6 Fig — Cells were first preincubated with either buffer alone or with buffer + EDTA 2.5 mM for 5 min. Then, after 30 s control measurement, either human serum (HS) (green trace), or HS-opsonized Ad3 (red trace) (A) or Ad5 (black and blue trace) (A,B) were added (black arrow). In (B), cells were pretreated with 2.5 mM EDTA or not, and exposed to HS-opsonized Ad5 (untreated cells: black curve, EDTA treatment: blue). Results are representative of 3 independent experiments. (TIF) [file ppat.1013504.s006.tif]

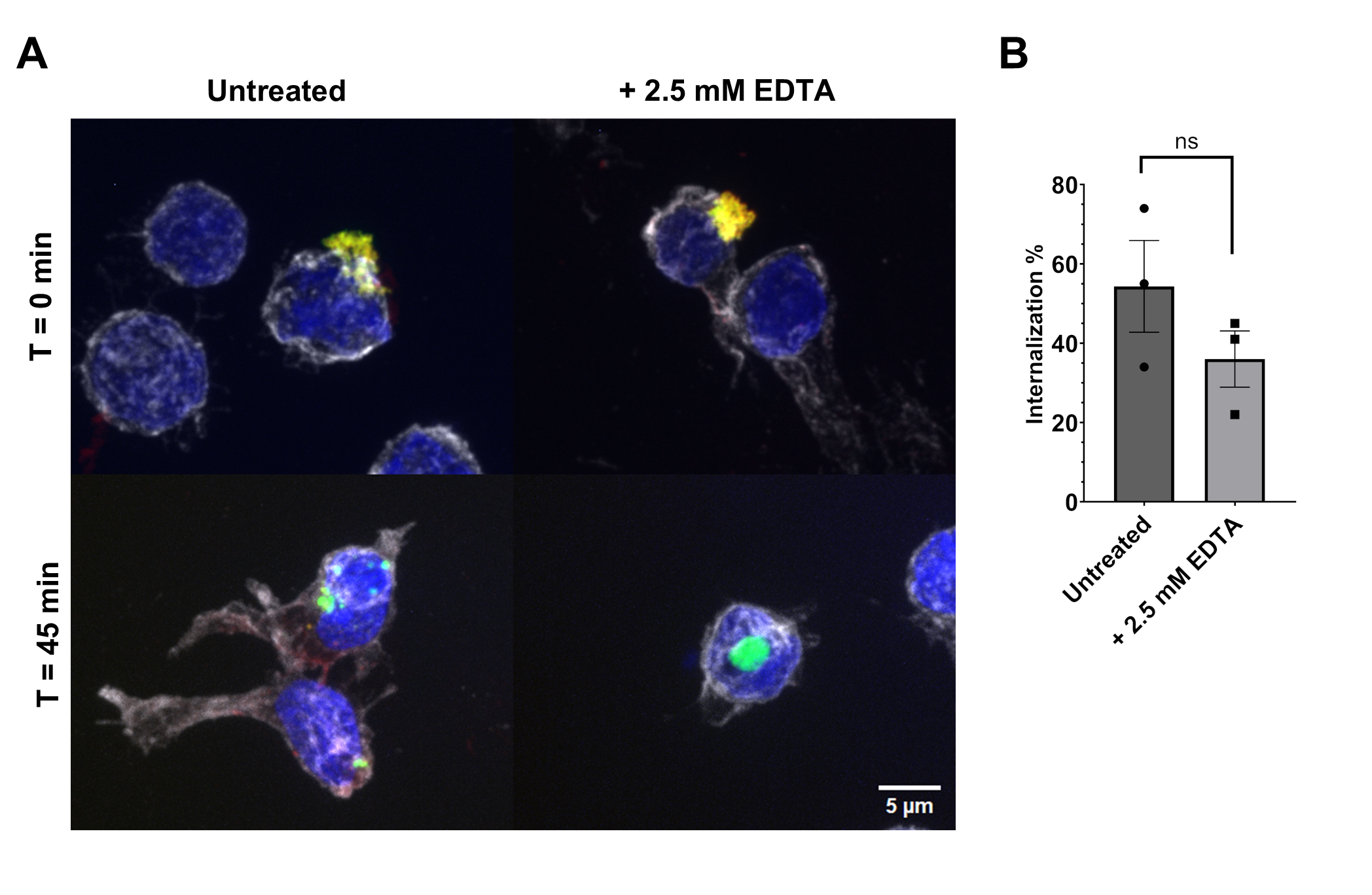

Supplement: S7 Fig — (A) PLB-985 cells were exposed to human serum-opsonized A-488 Ad5 (MOI 104 vp/cell) for 30 min at 4°C to allow binding then incubated for 0 or 45 min at 37°C. The left panel shows untreated cells while in the right panel, cells were pre-incubated with 2.5 mM EDTA. Extracellular A-488 Ads accessible to A-594 anti-IgG antibody are labeled in yellow (A-488-labeled and A-594 anti-IgG-labeled double positive particles) while intracellular Ads are only A-488-positive and appeared as green particles. The plasma membrane was stained with WGA CF-640R (gray) and nuclei were stained with DAPI (blue). (B) Quantification of the percentage ± SEM of internalized Ad patches after 45 min of incubation (3 independent experiments, n = 25 cells for the untreated condition, n = 24 for the condition with EDTA). Each dot represents the percentage of internalized Ad patches for each experiment. Mann-Whitney test was performed. ns, non significant. (TIF) [file ppat.1013504.s007.tif]

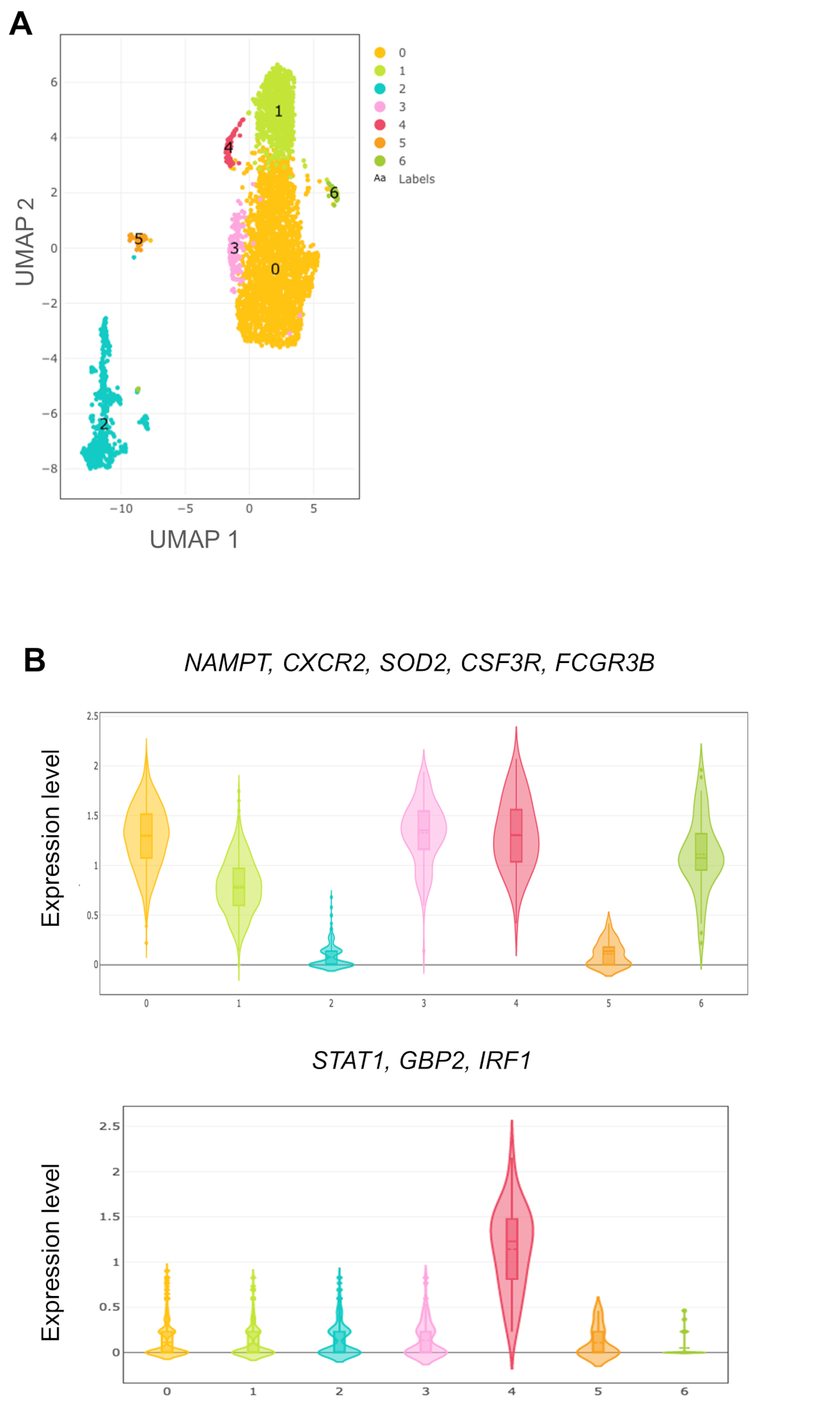

Supplement: S8 Fig — (A) Single-cell RNA-sequencing analysis of purified blood PMNs and incubated with either human serum (HS) or with HS-opsonized Ad5 for 1 h. Identification of 7 cell clusters using the SEURAT package. The clusters are visualized using Uniform Manifold Approximation and Projection (UMAP). Each dot represents one cell. (B) Upper panel: Log normalized expression level of NAMPT, CXCR2, SOD2, CSF3R, FCGR3B in the different clusters. Lower panel: as above for the interferon stimulated genes: STAT1, GRB2, IRF1. (TIF) [file ppat.1013504.s008.tif]

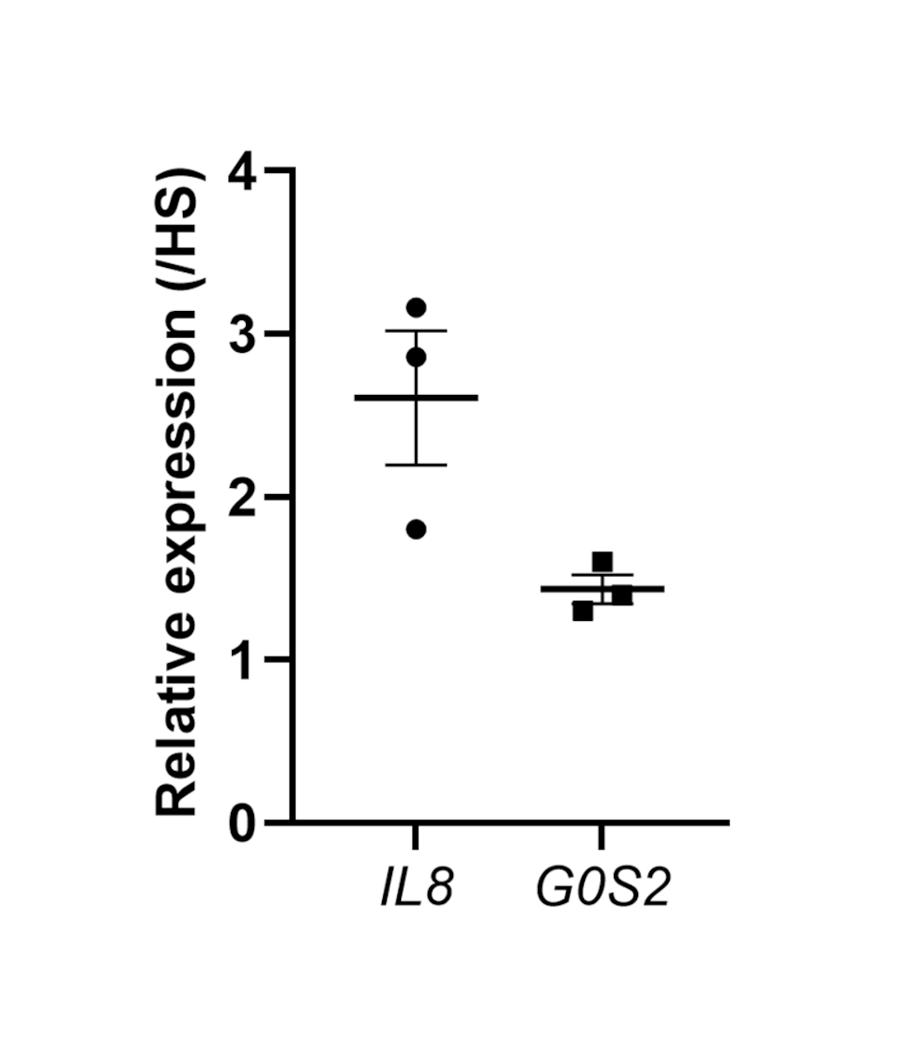

Supplement: S9 Fig — After normalization for GAPDH gene expression, the histogram shows the relative expression of CXCL8 and G0S2 mRNAs in PMNs in the presence of Ad5 as a function of the expression in cells incubated with HS alone. Data show the mean ± SEM and are from three experiments with technical duplicates for each condition in every experiment. (TIF) [file ppat.1013504.s009.tif]

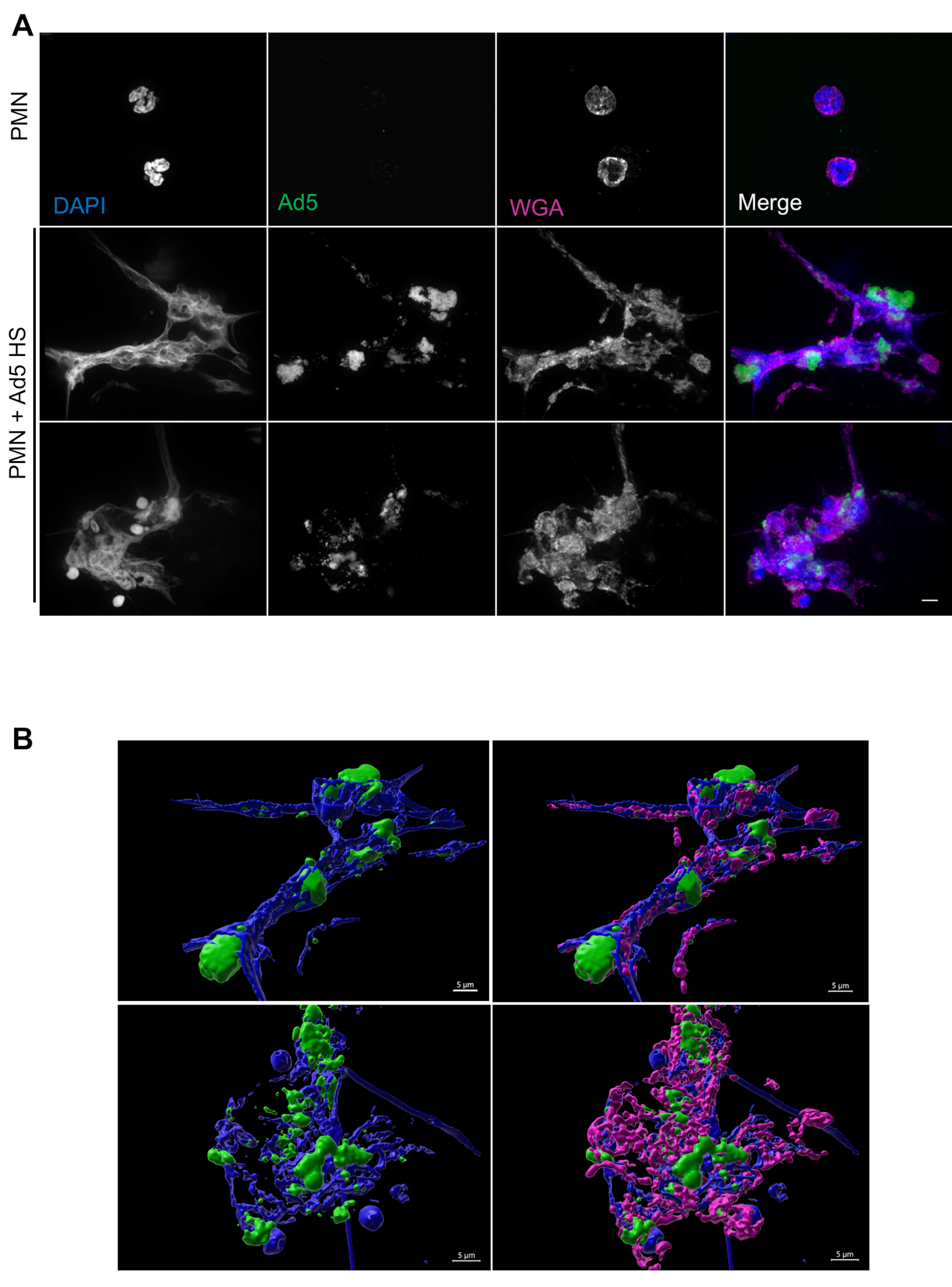

Supplement: S10 Fig — PMN were exposed or not to human serum-opsonized A-488 Ad5 (MOI 104 vp/cell) for 30 min at 4°C to allow binding then centrifuged and washed to eliminate unbound Ads. The cells were then incubated for 3 hours at 37°C. The upper panel shows PMNs alone and the lower panels PMNs incubated with HS-opzonized A-488 Ads (green). The plasma membrane was stained with WGA CF-640R (magenta) and nuclei were stained with DAPI (blue). (A) Representative images acquired by spinning disk confocal microscopy. Each image represents a projection of a Z stack. Scale bar, 5µm (B) 3D segmentation of the merge images of A (lower panels) to highlight NETs and Ad5 trapped in NETs. Scale bars, 5µm. (TIF) [file ppat.1013504.s010.tif]

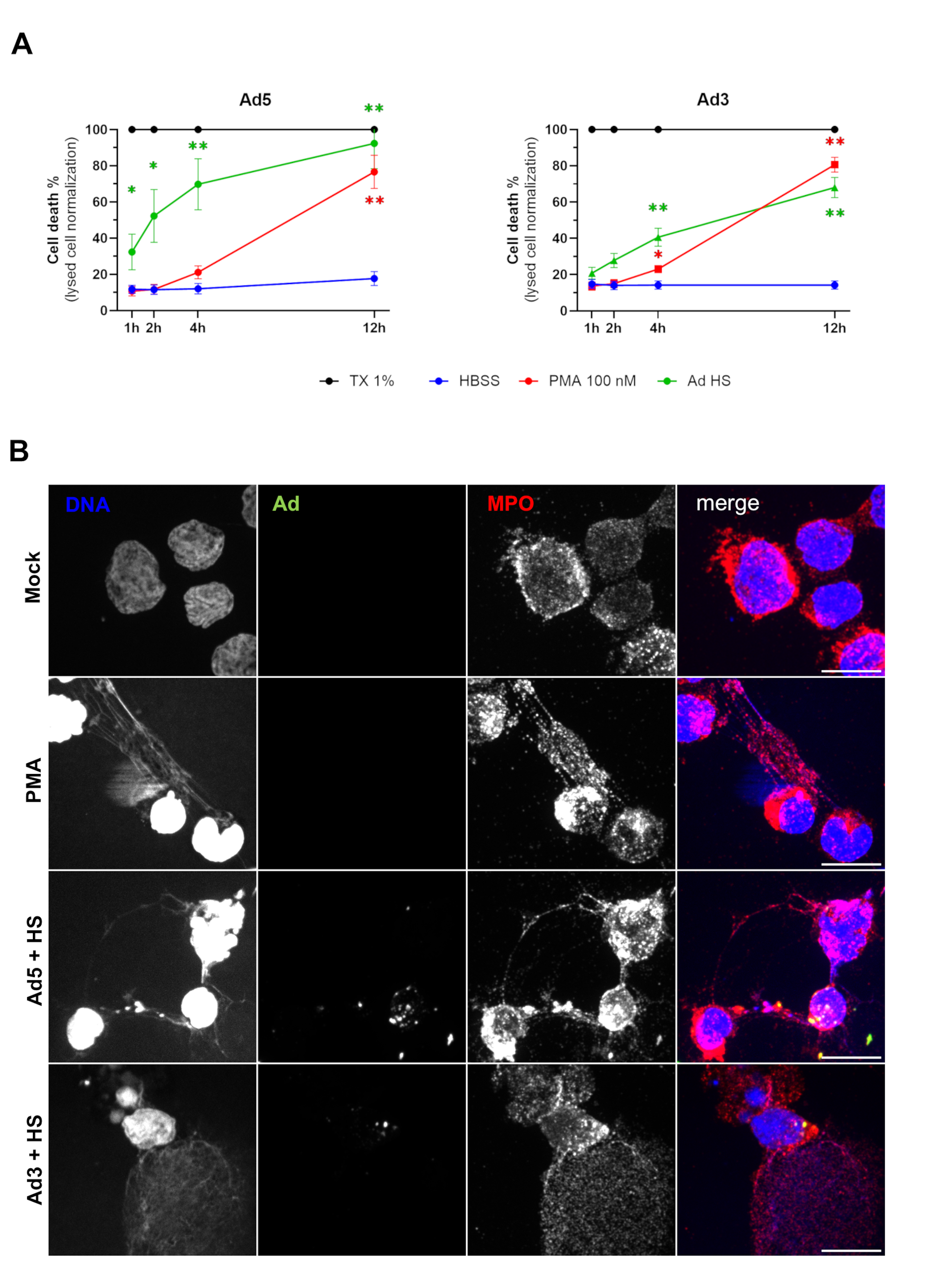

Supplement: S11 Fig — (A) Ad cytotoxicity assessed via Sytox Green assay. PLB-985 cells were incubated with HBSS, Triton-X-100 1%, or HS-opsonized Ads (MOI 104 vp/cell) in presence of Sytox Green probe, a cell-impermeant DNA fluorescent probe. Cell fluorescence was measured with a plate reader. The curves represent the cell death percentage (mean ± SEM, i.e., cell fluorescence relative to Triton-X-100-lysed cell fluorescence multiplied by 100) at different times post-infection.Mann-Whitney test was performed. *, p < 0.05; **, p < 0.01; (comparison with PLB-985 cells incubated with HBSS). Five independent experiments, with technical triplicates for each condition in every experiment, were performed. (B) Ad-induced NET (neutrophil extracellular trap) formation. PLB-985 cells were incubated with buffer (Mock), PMA 100 nM (positive control) or labeled and HS-opsonized Ad5 or Ad3 (MOI 104 vp/cell) for 4h, then fixed, permeabilized and stained for DNA (blue) and myeloperoxidase (MPO). Scale bar = 10 µm. Five independent experiments, with technical triplicates for each condition in every experiment, were conducted. (TIF) [file ppat.1013504.s011.tif]

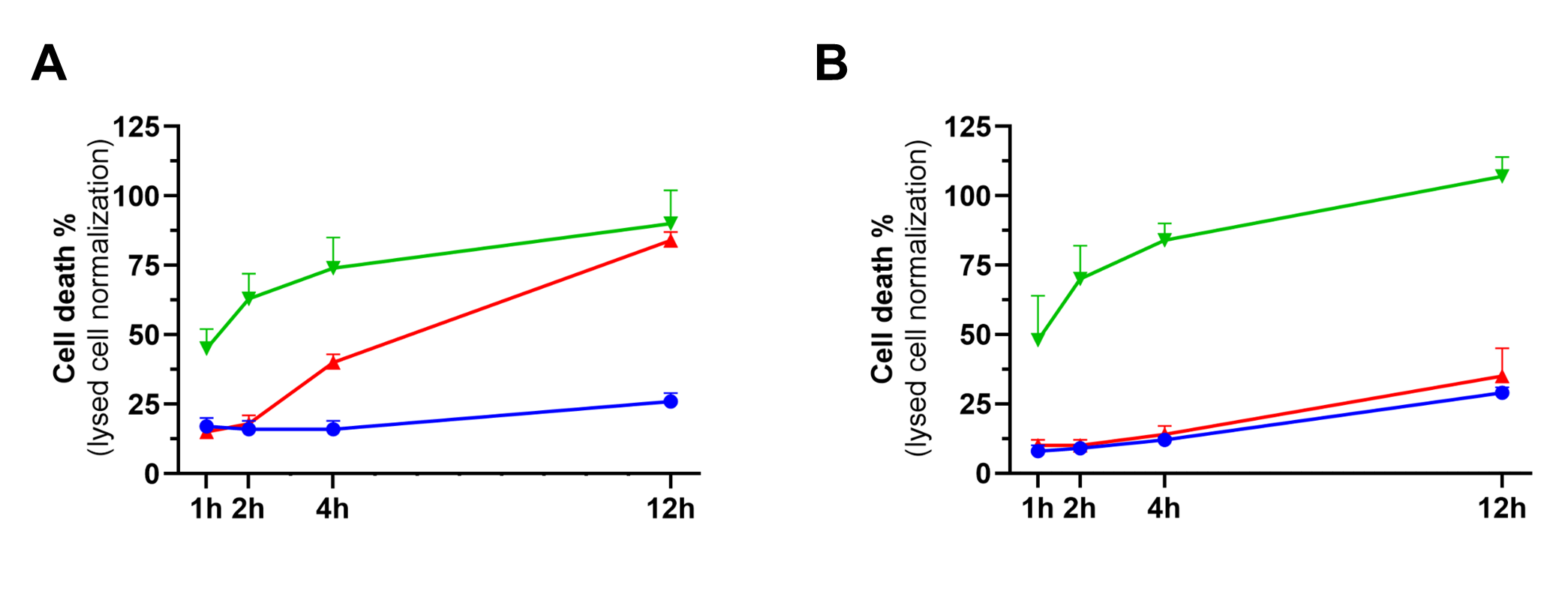

Supplement: S12 Fig — (A) PLB-985 WT cells were incubated with Sytox Green probe, then with HS (blue), HS-opsonized Ad5 (green) or PMA 100 nM (red). Sytox Green fluorescence was measured at the indicated time points and expressed relative to the cells treated with Triton-X-100 (+ SEM). (B) The same experiment was performed on NOX2-deficient (NOX2-/0) PLB-985 cells. Six (A) and four (B) independent experiments, with technical triplicates for each condition in every experiment, were performed. (TIF) [file ppat.1013504.s012.tif]

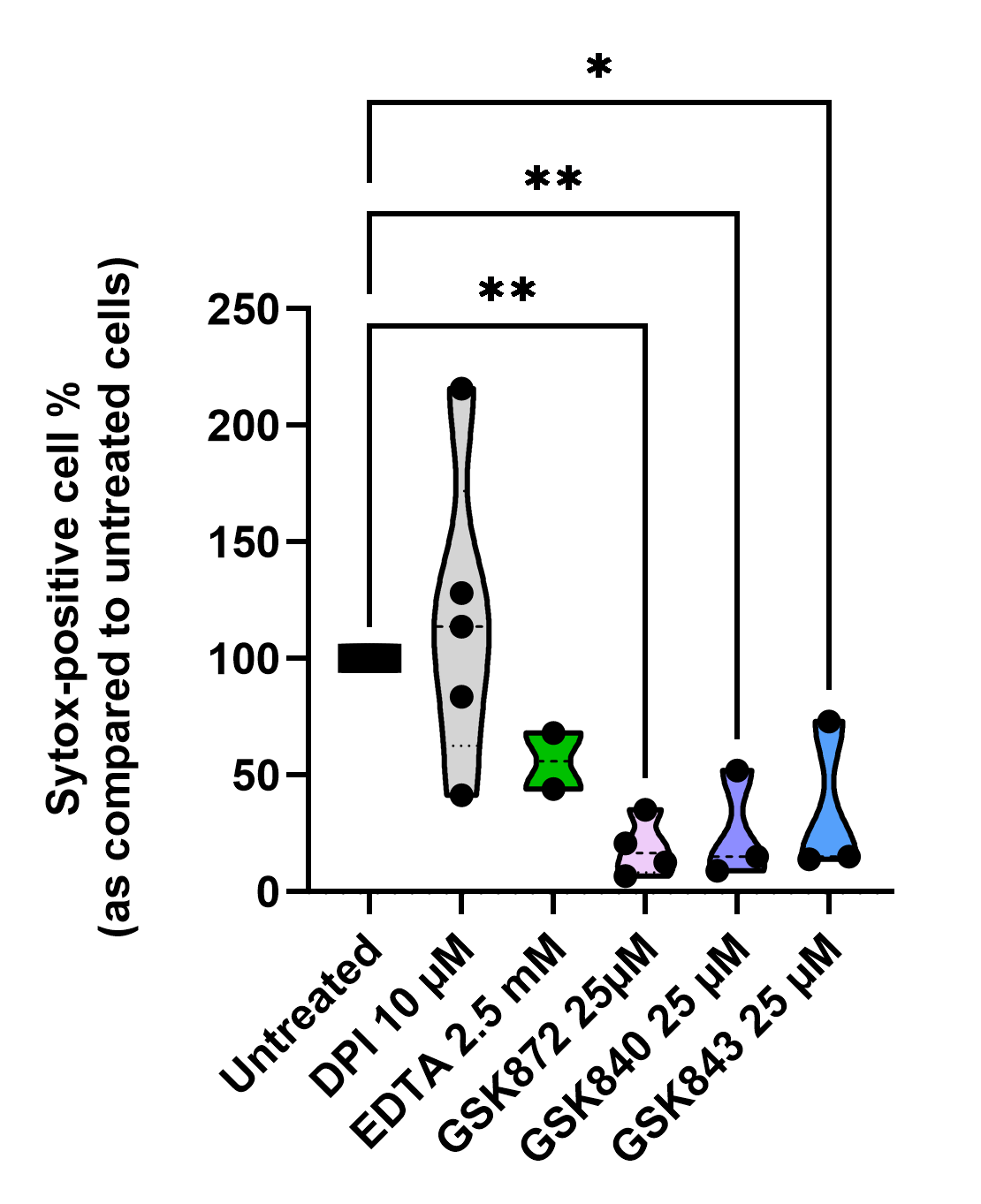

Supplement: S13 Fig — The same experiment as shown in Fig 7D was performed with Ad3. PMNs were pre-incubated with different inhibitors in the presence of Sytox Green probe and incubated with HS-opsonized Ad3. After 4h, Sytox Green fluorescence was measured and expressed as a ratio relative to the fluorescence of the Ad-exposed cells treated with the respective inhibitor or HS. The condition without inhibitors (untreated cells) was set at 100%. Kruskal-Wallis test was performed followed by Dunn’s multiple comparison tests: **, p < 0.01. Three independent experiments, except for EDTA condition (2 experiments), with three technical replicates per condition for each experiment were performed. (TIF) [file ppat.1013504.s013.tif]
