# Supplementary material for: Adenovirus phagocytosis by neutrophils triggers a pro-inflammatory response
Source: PLoS Pathog. 2026 Apr 6;22(4):e1013504. doi: 10.1371/journal.ppat.1013504 (PMC13102305; doi:10.1371/journal.ppat.1013504)
Supplement: S1 File — (DOCX) [file ppat.1013504.s014.docx]

## **Supplementary Material and methods.**

**Human serum anti-Ad ELISA**

After coating 96-well plates (Nunc) with 100 ng inactivated Ad5 or Ad3, the human serum used for Ad opsonization was diluted in serial dilutions in 5% milk PBS-Tween 0.05% and incubated 1h at room temperature. Bound antibodies were detected with peroxidase-conjugated goat anti-human IgG (Southern Biotechnology, 2040-05, 1:5000) antibody after 1h incubation at room temperature. The peroxidase activity was revealed by incubation with the substrate O-phenylenediamine dihydrochloride (Sigma–Aldrich) for 30 min. The reaction was stopped by addition of 3 N HCl and optical density (OD) measurement was performed at 490 nm. Titers were defined as the highest dilution giving an OD 490nm 2-fold above background values.

**Extracellular and intracellular Ad detection**

The protocol was adapted from Berhens *et al.* (69). Cells (5 × 10^5^) were resuspended in HBSS solution then plated on polylysine-coated glass coverslips for 30 min at 37°C. HS-opsonized A-488nm labeled Ads were then added on PLB-985 cells (MOI 10^4^ vp/cell) pre-treated or not with EDTA 2.5mM. Then, the cells were incubated at 4°C with or without a further incubation at 37°C for 45 min. After PFA fixation, Fc receptors were blocked as described previously then the plasma membrane was labeled with CF 640R WGA (Wheat Germ Agglutinin) (5 µg/mL final concentration) and the cells were incubated with anti-IgG A-568 nm antibody (Thermofisher A21090, 1:500) to label extracellular Ads. Finally, samples were permeabilized and then mounted on glass slides with DAPI-containing Fluoroshield mounting medium (Sigma-Aldrich, F6057).

**Neutrophil extracellular traps**

The protocol described in the manuscript was modified as described below to observe Ad trapped in NETs. After incubation of the PMN at 4°C with or without Ad5, the plates, containing the cells, were centrifuged at 120g for 4 min and after a washing step, they were incubated for 3 hours at 37°C. After a fixation step, Fc receptors were blocked as described previously then the plasma membrane was labeled with CF 640R WGA (Wheat Germ Agglutinin) (5 µg/mL final concentration). The cells were permeabilized and the samples were mounted on glass slides with DAPI-containing Fluoroshield mounting medium (Sigma-Aldrich, F6057).
