# Supplementary material for: Adenovirus phagocytosis by neutrophils triggers a pro-inflammatory response
Source: PLoS Pathog. 2026 Apr 6;22(4):e1013504. doi: 10.1371/journal.ppat.1013504 (PMC13102305; doi:10.1371/journal.ppat.1013504)
Supplement: Attachment — Submitted filename: Response to reviewers_VF.pdf [file ppat.1013504.s017.pdf]

Dear Editors,

We are pleased to resubmit our manuscript entitled "Adenovirus phagocytosis by neutrophils triggers a pro-inflammatory response" after addressing the reviewer's comments. We appreciate the constructive feedback provided by the reviewers and the editorial team, which has significantly improved the quality of our work.

In this revised version, we have carefully incorporated suggested modifications and provided detailed responses to each comment. Key revisions include:

- Improved microscopy images (fig. 2, 3, 4, 6) and additional experiments to better visualize Neutrophil Extracellular Traps (NETs) (S10 fig.),
- New experimental data confirming the role of RIPK3 in neutrophil death, strengthening our conclusions (fig. 7 and S13 fig.),
- Supplementary Figures for some experiments that were previously indicated as "data not shown" (S6 and S13fig.),
- Modified graphs to show all individual data points,
- Modified figures to make them clearer (fig. 6A and S11A,)
- Supplementary data in the "Response to Reviewers" document, some from new experiments, to justify our responses,
- Expanded discussion and references on Ad5 interactions with other immune cells,
- Emphasized the novelty of our study and clarifying its distinct contributions to the field,
- Supplementary files with all the experimental data for the graphs(raw data folder).

We believe these revisions strengthen the manuscript and align it more closely with the journal's scope and standards. We would be grateful for the opportunity to have our work reconsidered for publication in *PLoS Pathogens*.

Thank you for your time and consideration. Please let us know if any further information is required.
Sincerely,
Sophie Dupré-Crochet
(On behalf of all authors)

Journal Requirements:
1) We ask that a manuscript source file is provided at Revision. Please upload your manuscript file as a .doc, .docx, .rtf or .tex. If you are providing a .tex file, please upload it under the item type u2018LaTeX Source Fileu2019 and leave your .pdf version as the item type u2018Manuscriptu2019.

A manuscript source file corresponding to the revised version of our manuscript was uploaded.

2) We noticed that you used the phrase 'data not shown' in the manuscript. We do not allow these references, as the PLOS data access policy requires that all data be either published with the manuscript or made available in a publicly accessible database. Please amend the supplementary material to include the referenced data or remove the references.

The supplementary material was modified to include now all referenced data.

3) We do not publish any copyright or trademark symbols that usually accompany proprietary names, eg ©, ®, or TM (e.g. next to drug or reagent names). Therefore please remove all instances of trademark/copyright symbols throughout the text, including:
- ® on page: 19.
- TM on page: 23.

This was done as requested.

4) Please upload all main figures as separate Figure files in .tif or .eps format. For more information about how to convert and format your figure files please see our guidelines: https://journals.plos.org/plospathogens/s/figures

All main figures were uploaded as requested.

After careful consideration, we feel that it has merit but does not fully meet PLOS Pathogen's publication criteria as it currently stands. Therefore, we invite you to submit a significantly revised version of the manuscript that addresses the points raised during the review process by reviewers 2 and 3. Please pay particular attention to the points raised by Reviewer 2 about submitting higher quality and more compelling microscopy images. Please also address the significant critiques raised by Reviewer 3 about both the technical and conceptual novelty of your study.

Reviewers' Comments:

Reviewer's Responses to Questions

Part I – Summary

Please use this section to discuss strengths/weaknesses of study, novelty/significance, general execution and scholarship.

Reviewer #1:
Laurans et al revisit human adenovirus phagocytosis by neutrophils. The last paper in this area seems to have been published in 2005 and the progress in tools and reagents over the last 20 years certainly justifies a revisit with new technology.

The authors use fluorescently labelled Ad3 or Ad5 to infect primary human neutrophils or PLB-985 cells at an MOI of 10,000 (same MOI as Cotter et al in 2005). Similar to the former study, this study confirms that Ad association with neutrophils requires IgG recognition and CD32 and results in internalization. Using confocal microscopy and TEM, they confirm that virus is located in membrane delimited compartments that colocalize with Rab5 and CD63, markers of the phagosome and triggers NOX2 dependent ROS production that is dependent on extracellular calcium.

The novelty of the current submission is related to single cell RNA sequencing reveals that Ad interaction triggers a unique transcriptional response, including upregulation of CXCL8. In addition, this study goes on to investigate the outcome of Ad interaction with PMNs, showing that exposure to Ad triggers rapid PMN death, that is RIPK3 dependent but not ROS dependent, and releases NETs and CXCL8. Thus, Ad induced PMN death appears related to "necroptosis". Additional data, suggests that phagocytosed Ad were not killed and remain functionally able to infect target susceptible cells.

Overall, I thought this was a nice extension of the Cotter paper from 2005 that builds on those observations to show that Ad engulfment by PMNs leads to changes in transcriptional programs and rapid killing of PMNs. This work also begins to parse out the mechanism of how this is achieved.

Reviewer #2:
Comments to the submission by Laurans et al., Adenovirus (AdV) phagocytosis by neutrophils triggers a proinflammatory response.' Immune cell interactions with viruses drive both anti-viral defense and pathogenesis. In this context, AdV neutrophil interactions have not been systematically explored, and much is to be discovered. AdV are pathogens of vertebrates, and in humans they cause a variety of disorders ranging from respiratory and gastrointestinal tract infections to acute viremia with systemic effects, often followed by viral persistence for years. Previous studies of AdV interactions with transformed and nontransformed cell lines and primary cells, including dendritic cells, natural killer cells and macrophages, set the stage for the type of work presented here with neutrophiles.

The authors apply conventional cytometry, bulk RNAseq and some single cell light and electron microscopy to describe interactions of two human AdVs, AdV-C5 and AdV-B3 with human neurophils. Their results show that polymorphonuclear neutrophils (PMNs) or neutrophile-like cells undergo cell death within a few hours after inoculation with immunocomplexed Ad5 or Ad3. Unfortunately, the study is descriptive, and does not provide molecualr mechanisms for AdV triggered neutrophile death.

General remarks

In the current version of this mansucript, reference to the literature is not sufficient, and the critical points brought up in introduction and discussion sections are sparsely supported by references and should be enriched with more contextual information. For example, AdV interactions with immune cells and the cell response thereupon, including virus cell entry and gene delivery as well as innate detection by host DNA sensors along with cytokine signalling have been described before in primary cells as well as cell lines, such as THP1. Notably, it had been shown earlier that AdV targeted to Fc receptors penetrates to the cytosol and delivers genes to the cell nucleus, along with being sensed by cGAS triggering the expression of a range of cytokines and chemokines. It would be important to crossreference to the data from previous reports on AdV interactions with other immune cells, and also discuss why there is no AdV present in the cytosol of the PMNs and PLB-985 cells. This seems to be critical to block viral gene expression and antagonism of neutrophile cell death.

Recommendation
To reach par with previous studies on AdV host cell interactions and allow for comparision of AdV infection of other cell types based on previous studies the authors should better characterize AdV induced neutrophil death, Ca influx, CXCL8 release and especially also the neutrophil extracellular trap (NET) formation process. This would potentially make this study an important and valuable contribution. Besides small chemical inhibitors used already, further experiments should include perturbations with RNA interference, use of virus mutants impaired in gene delivery, UV inactivated inocula as well as critical dose-response experiments to assess specificity and sensitivity. Further details see below.

Reviewer #3:
The manuscript by Laurans et al examines the interaction of adenoviruses (Ad3, Ad5) with neutrophils. The authors examine adenovirus binding to neutrophils followed by phenotypic effects including calcium mobilization, ROS formation, transcriptomics, CXCL8 release, cell death, neutrophil extracellular trap formation, and viral resilience. The study is done completely in vitro and relevance to anti-adenoviral immunity in vivo is unclear. The first part of the paper recapitulates a lot of work

performed in the field more than 20 years ago. The newer aspects of the study are not conducted in sufficient depth, and data are not tied together into a unified conceptual framework.

Part II – Major Issues: Key Experiments Required for Acceptance
Please use this section to detail the key new experiments or modifications of existing experiments that should be absolutely required to validate study conclusions.

Generally, there should be no more than 3 such required experiments or major modifications for a "Major Revision" recommendation. If more than 3 experiments are necessary to validate the study conclusions, then you are encouraged to recommend "Reject".

Reviewer #1:

1)I am not sure this is a major issue. However, I find it hard to reconcile how infection at the incredibly high MOI of 10,000 (same as Cotter used), which leads to over 50% of cells associated with Ad and also induces 50% of cells to die by 4 hrs post exposure, lead to cluster 6 (those associated with Ad) being only 1% or less of the cells on the UMAP shown in Fig. S8. Some explanation should be provided for this disconnect, which seems like a major inconsistency.

We agree with the reviewer that the percentage of cells identified as neutrophils was low for the condition with adenoviruses (Ads). There are two non-mutually exclusive possibilities to explain this observation:

1. **Differential UMAP Clustering:** The single-cell software, trained on Blueprint Epigenomics and Encode database mRNA profiles, identified cells from clusters 0, 1, 3, 4, and 6 as PMNs, cluster 5 as T lymphocytes, and did not manage to identify cluster 2 (S8A Fig.). This latter cluster, which is composed mostly of cells exposed to Ad5, could represent a specific population of neutrophils or could be lymphocytes. We cannot know.
2. **Cell aggregation and Death:** We may have failed to retrieve isolated PMNs because PMNs exposed to adenoviruses (Ads) aggregated (fig 6B and S10 fig.) and died. PMN aggregation has been described by others; for example, PMNs exposed to zymosan aggregate [1]. For the single-cell RNA technique, aggregates are discarded as described in the "Materials and Methods" section, leading to the loss of PMNs at this stage. Moreover, as shown in Figure 6, 30% of neutrophils incubated with Ads already died after 90 minutes.

2) This is really a minor issue, but not as minor as those listed below. It would be nice to know how far the pH changes in the endosome during Ad internalization before PMN death. Given that the virus remains infectious, it can't drop far enough to cause capsid disassembly. Is there some interference with acidification? Some discussion on this point would be warranted, if not an experiment.

The phagosomal pH of PMNs has been described as less acidic compared to that of other phagocytes. Jankowski *et al.* evaluated this phagosomal pH during the first 40 minutes following phagocytosis and demonstrated that the phagosomal pH remains neutral [2]. This could explain why we may not observe capsid disassembly.

3) I find the final section on the ability of the Ad to remain infectious after phagocytosis and PMN death to be very interesting, but not well developed. In theory, at least some of the IgG in pooled human serum would be expected to be "neutralizing" and block infection. The fact that Ad that has passage through PMN phagocytosis remains infectious is very interesting and a potentially exciting observation. Is it possible that the phagocytosis process removes neutralizing antibody, actually reactivating IgG neutralized virus? This would be a VERY good trick for a virus, but a new assay would have to be set up

to see if infectious virus could be recovered from fully IgG neutralized virus by passage through PMNs. Some discussion on this point would be warranted, if not an actual experiment.

The reviewer raises a very interesting point. We did immunofluorescence experiments using an anti-human IgG antibody to detect if the virus was still covered with IgG after internalization. We first incubated Ad5, labelled with Alexa488 and pre-incubated with human serum, with the neutrophils at 4°C to favor the binding then removed all the unbound Ad5 by centrifugation and washing. The cells with bound Ad5 were then incubated 15min or 3h at 37°C. The anti-human IgG antibody was incubated after fixation before permeabilization to detect extracellular Ad5. WGA labelled with CF640R was then added to stain the plasma membrane as explained in the supplementary method in the paragraph "Extracellular and intracellular Ad detection". We observed that Ad5 could be marked or not by the anti-human IgG antibody (Figure 1 below), so we may have a partial release/destruction of the IgG. These data are preliminary and should be confirmed by other experiments.

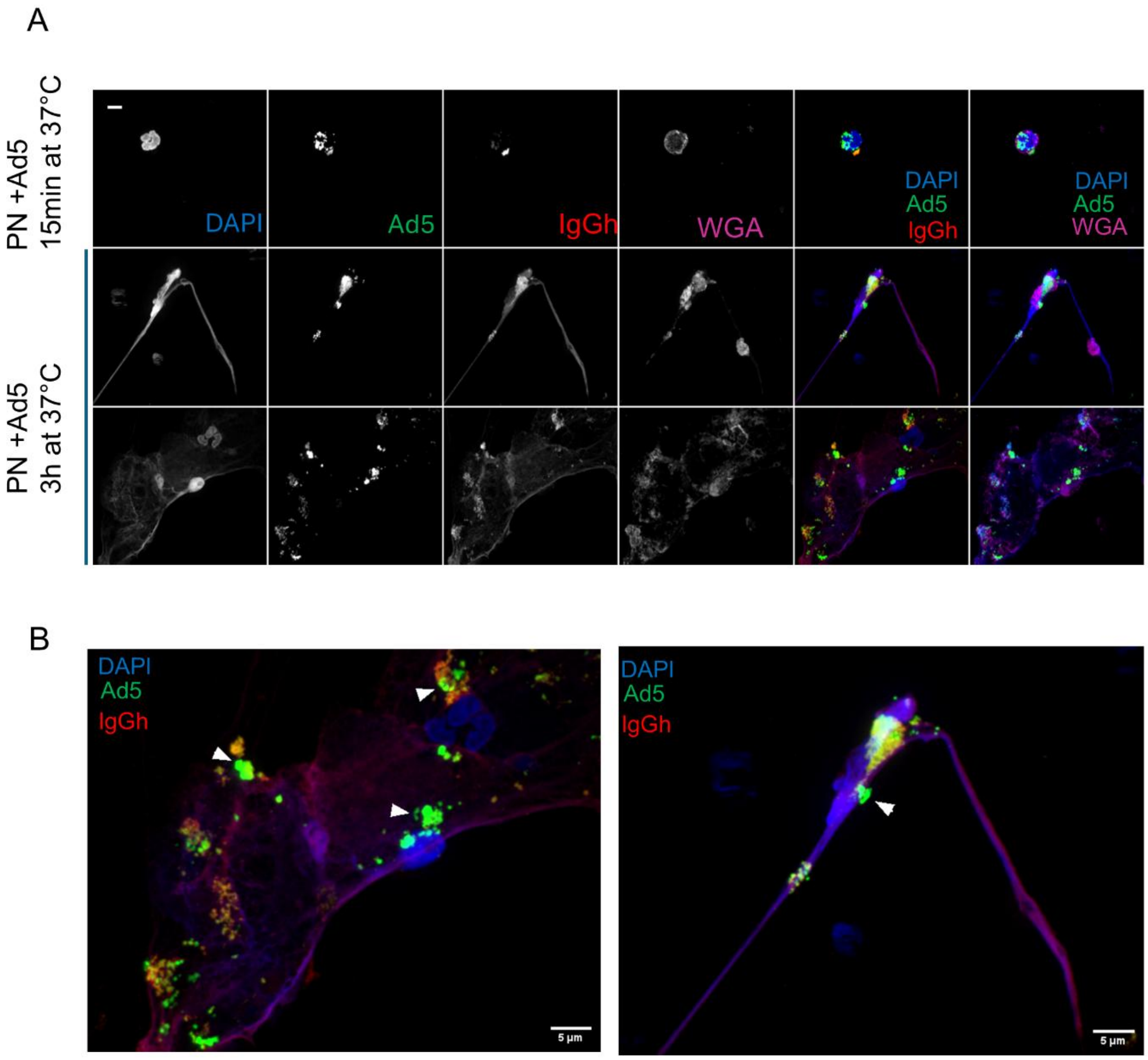

Figure 1: Representative Images of PMN incubated with Ad5 after 15min or 3h at 37°C.
(**A**) The upper panels are representative images of the PMN incubated with Ad5 for 15min at 37°C, the two other panels are representative images of the PMN incubated with Ad5 for 3h at 37°C. The nucleus was stained with DAPI (blue), Ads5 were labelled with A488 (green), the antibody against human IgG

was labelled with Alexa 578 (red) and the plasma membrane was stained with WGA labelled with CF640R (magenta). (**B**) Enlarged images of PMN incubated with Ad5 for 3h at 37°C showing the NETs and Ads5 marked or not (arrows) by the anti-IgG antibodies.

Reviewer #2: Specific points

1) The quality of the immunofluorescence images is generally low. Representation of these data needs to be improved throughout the study. For example, even the DAPI signals of control cells appear to be out of focus (see, e.g., Fig. 2, 3, 4, and in part Fig. 6).

We made again the figures cited above starting from the raw images. The .tif files of the figures are joined.

2) The annotation of the figures, including images is insufficient. The composites are not self explanatory and should be improved.

We enhanced the annotation by indicating on the images which colors correspond to which signals.

3) Line 95: Ref 18 is for Ad7 not Ad3. Please amend.

We thank the reviewer for pointing out this mistake. We modified the reference.

4) Fig 1A: What is an association index? I guess these are some normalized fluorescence units of virus particles, but it is unclear if these signals are composed of virus aggregates or monodispersed particles. Please provide fluorescence images.

As explained in the results section, the association index corresponds to the mean fluorescence intensity multiplied by the percentage of Alexa 488-labeled Ad-positive cells. We mostly observe virus aggregates. Measurements of the Ad signals in different immunofluorescence experiments, either immediately after binding or 10 minutes after incubating the cells at 37°C (Fig. 2, 3A), indicate sizes of 0.9 $\mu m^2 \pm 0.2$ for Ad3 patches and 2.3 $\mu m^2 \pm 0.5$ for Ad5. Given that the point spread function (PSF) for this optical system is 0.250 μm^2, we would expect monodispersed particles to yield a signal close to this PSF value. Since this is not the case, we can assume that the Ad signals are predominantly composed of virus aggregates. This can be explained by the fact that viruses are crosslinked by the antibodies.

5) Line 128: Another possibility is a different amount of monodispersity of the inocula. To control for the quality of the fluorescently labeled Ad5 and Ad3 inocula, the authors should run scatter analyses or fluorescence imaging of the labeled virions.

We measured the polydispersity index (PDI) of the labelled Ad by Dynamic Light Scattering and find a PDI comparable for the two labelled Ad and for beads of the same diameter.

6) Line 130: please check / comment on the presence / absence of the high affinity Fc receptor CD64 in the cells used in this study. If the high affinity CD64 Fc receptor is detected by flow cytometry, e.g., then its effects on Ad-immunocomplexes should be tested, given that anti-CD32 and anti-CD16 antibodies had only small effects on cell association of the immunocomplexes.

We did not check the effect of CD64 because the high-affinity Fc receptor is expressed only when neutrophils are stimulated with IFN-γ and GM-CSF [3,4]. Since we did not stimulate the PMNs, they primarily expressed the CD32 and CD16 receptors. So we checked the involvement of these two receptors.

7) Fig. 2: It seems as if only a minority of cells binds immunocomplexed Ad3 or Ad5. It is not clear how representative these data are. Please provide better resolved images, and quantifications of single cell analyses.

We measured Ad binding using flow cytometry. Fig. 1A shows that the association index (mean fluorescence intensity multiplied by the percentage of Alexa 488-labeled Ad-positive cells) is low for both Ad3 and Ad5. However, the association index increases by 150 for immunocomplexed Ad5 and by 20 for immunocomplexed Ad3 (Fig. 1B). As shown in Figure S3, at least 50% of the cells are positive for immunocomplexed Ads. We measured binding by flow cytometry and assessed internalization by confocal microscopy.

8) What is the mechanism of cell death of the neutrophile-like cells? Why does the NADPH oxidase (NOX2) inhibitor DPI inhibit cell death triggered by PMA but not immunocomplexed AdV?

The mechanism of neutrophil cell death depends on the nature of the stimuli [5]. We do not know why NADPH oxidase does not trigger neutrophil cell death. However, PMA induces a very strong activation of NADPH oxidase, which is not the case with Ads. We found that Ads trigger NADPH activation that is 5 to 6 times lower than that induced by opsonized zymosan (yeast cell wall fragments), as shown in Figure 2 below. Opsonized zymosan, in turn, triggers a lower activation of NADPH oxidase than PMA [6]. Therefore, the amount of ROS produced may not be sufficient to trigger NETosis which can also occur through alternative pathways. Indeed, Kenny *et al. reported a* NADPH oxidase (NOX)-independent NETosis induced by calcium ionophore [7].

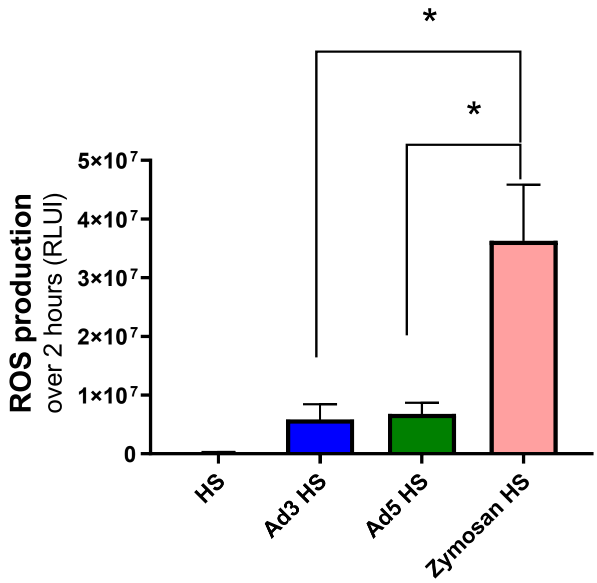

Figure 2 : ROS production. PMNs were incubated with HS, HS-opsonized Ad (MOI 10^4 vp/cell) or zymosan (MOI 10 particles/cell). The graph shows the integrated ROS production over 2 hours, detected by a luminometry-based test from four independent experiments. Two technical replicates were performed for each experiment. Statistical significance determined by Mann Whitney test. **, $p < 0.05$.

9) There is a substantial risk for off-target effects of DPI, including cell tox. What was the minimal concentration of DPI necessary to inhibit ROS production and cell death? Please provide dose-response curves here, at least for the PMNs and PLB-985 cells. The latter lack NOX2 and should not be subject to on-target effects of DPI.

DPI has been commonly used for NADPH oxidase (NOX) inhibition at a concentration of 10 µM in various studies (e.g., [8]). It has been shown that at this concentration, DPI inhibits ROS production by the NADPH oxidase but not mitochondrial ROS production [9]. Some off-target effects have been noted in non-immune cells but at higher concentrations (10-fold higher) [10].

To verify the effect of ROS on cell death, we used the NOX2-deficient PLB-985 cell line (S11 Fig.). The wild-type PLB-985 cell line produces ROS (S5 Fig., [11]).

10) Line 171: data not shown. Please provide the data.

We added the data in the S6 Fig. for the PLB-985 cells with Ad3 and Ad5.

11) Line 173: Cells treated with the Ca chelator EGTA did not exhibit Ca increase in the cytosol upon application of immunocomplexed AdV. The authors interprete this to mean that Ca is not released from internal stores in AdV inoculated cells, also in absence of EGTA. This stands on weak grounds. How can the authors exclude the possibility that Ca chelation depletes the internal Ca stores that would normally respond to immunocomplexed AdV?

EDTA at the concentration used (i.e. 2,5mM) largely depletes extracellular calcium and we could measure using the Bad4 software that the remaining free calcium concentration is 10µM. The concentration of the cytosolic calcium didn't change 15 min after EDTA addition as compared to the control condition: in the first 30s of the calcium measurement, before adenoviruses addition, the cytosolic calcium concentration is around 80nM (Fig. 3 below) as for non-EGTA-treated cells. In these conditions, EDTA is not able to induce the depletion of the cytosol, and therefore of the ER.

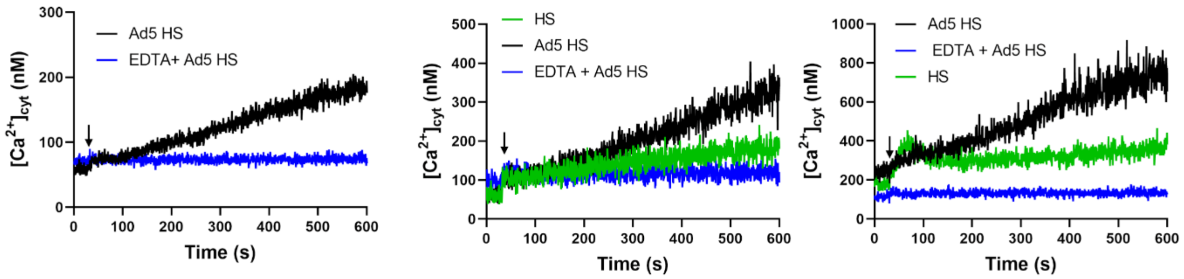

Figure 3 : Cytosolic calcium concentration in PLB 985 cells (left and middle panels) and in PMN (right panel). Human serum and adenoviruses were added 30s after the beginning of calcium measurement (arrow).

12) A related follow-up question is: What is the mechanism of Ca influx in these experiments? There is evidence in the literature that AdV entry into cells triggers Ca transients involving extracellular Ca from the medium via pores in the plasma membrane. Is there a similar mechanism at play here?

We have evidence that it involves calcium channels and their characterization will be the objectives of a new work.

13) Fig. 5, regarding transcriptome. It is interesting to note that the genes G0S2 and CXCL8 were upregulated in cells treated with immunocomplexed AdV. Unfortunately, these observations are not followed up. Please amend.

We added some sentences about *CXCL8* and its upregulation in the discussion (lines 322-24 in the manuscript file). We have already discussed about *GOS2* (lines 325-330) and highlighted the fact that both genes are upregulated in PMNs of patients at high risk of septic shock (line 337).

14) Fig. 6B: The images available to this reviewer are not of sufficient quality to unequivocally distinguish NETs from nuclei. Can the authors provide nuclear envelope marker stainings to distinguish the bona fide nuclei from the NETs?

We did new experiments using a membrane marker (WGA labelled with CF640R). Representative images in S10 Fig. illustrate the presence of neutrophil extracellular traps (NETs). NETs are clearly distinct from nuclei.

15) Fig. 7B: GSK872 used at 25 uM inhibited cell death. At such high concentrations, GSK872 will have off-target effects. Other RIPK3 inhibitors along with dose response curves and RNAi should be used to critically assess the GSK872 results.

We used two other RIPK3 inhibitors that confirmed the results with GSK872 inhibitor. The data are presented in Fig. 7E et S13 Fig.

16) Fig. 8A: missing annotation of the different bars.

We added the legend for the left panel.

17) Line 260: The inhibition of the CXCL8 receptor by reparixin did not inhibit PLB-985 cell death triggered by AdV. Please amend.

We added an explanation to this point (line 268-270 in the manuscript file).

18) Line 503: this must be a wrong reference. Please correct.

We corrected this mistake and added the right reference.

Reviewer #3:

1) The paper lacks focus and context. What is the importance of neutrophils and antiviral immunity? What is the central premise of the paper? Adenovirus binding to neutrophils? Adenovirus-induced neutrophil death? Adenovirus induced NET formation? The study touches on all of these subjects, but only superficially and the data are not tied together to create conceptual unity. Thus no real conclusions can be drawn.

Neutrophils are the main circulating leukocytes (40-60%) and the first cells recruited at injury or infection site thus constituting the first line of innate immune defense. They play an ambivalent role in viral infections since, depending on the infecting virus, they can help for the infection resolution or on the opposite, being excessively recruited and/or activated, leading to host tissue damage. Given that neutrophils have only recently been studied in depth, understanding their influence on antiviral immunity is essential.

The primary objective of this study was to investigate the interaction between neutrophils and adenoviruses, with particular focus on the consequences of this interaction for neutrophil function. Our key findings demonstrate that:

1. Neutrophils actively phagocytose adenovirus particles, confirming previous observations while extending our understanding of this process,
2. This phagocytosis event triggers significant biological responses, including:

-The release of IL-8, suggesting activation of inflammatory pathway,

-Induction of neutrophil cell death, representing a novel consequence of adenovirus interaction,

3. We have initiated mechanistic investigations into the cell death pathway, proposing a working model depicted in Fig. 10 of the manuscript.

This work represents an important advancement in our understanding of how adenoviruses interact with innate immune cells, particularly through their ability to induce neutrophil death and

inflammatory cytokine production. The proposed mechanism in Fig. 10 provides a framework for future studies to further elucidate these processes.

2) The adenovirus binding studies are done at 4 degrees C. The relevance of these findings is questionable. Studies should also be performed at 37 degrees to determine if viral internalization is also impacted by other cell receptors and pathways.

To identify the pathways involved in adenovirus binding to neutrophils, we performed flow cytometric analysis of virus binding at 4°C to specifically assess receptor-mediated interactions without internalization. For subsequent experiments examining viral internalization and post-entry effects, neutrophils were synchronized and then incubated with adenovirus at 37°C to permit active uptake.

3) The human serum used in the study was purchased from Sigma, and from male donors. Experiments should be performed with fresh serum/plasma from both male and female donors.

We confirmed the presence of antibodies against Ad3 and Ad5 in the Sigma serum (S2 Fig.) and observed corresponding binding and phagocytosis events. While we acknowledge that fresh serum might have provided more robust results, we deliberately chose to use the same commercial serum throughout all experiments for several reasons:

1. Using identical serum conditions across all experiments ensures comparability of results,
2. Commercial serum provides a standardized reagent that can be obtained by other researchers,
3. The Sigma serum offers convenient availability and batch consistency.

This approach allows for more reliable interpretation of our findings regarding adenovirus-neutrophil interactions while maintaining experimental rigor.

4) The relevance of the transcriptome studies is questionable. First, it does not make a lot of sense to do single cell transcriptomics on a homogenous population of neutrophils in vitro. Second, neutrophil effector functions are not normally transcriptionally regulated, and many of the genes (aside from CXCL8) have no relevance to the central premise and other observations of the paper. For example, what is the point of highlighting GOS2, a gene possibly involved in apoptosis when a) early apoptosis is generally a post-translational event and b) the authors show that neutrophils do not predominantly die by apoptosis. Furthermore, the transcriptome experiment was done at 90 minutes, barely enough time for all transcription events to occur.

Recent single-cell RNA sequencing studies have demonstrated that neutrophils constitute a heterogeneous population (ref 25, 26 of the article), a finding that aligns with our observations as discussed in lines 190-195 of the manuscript file. Our transcriptomic analysis identified several genes upregulated in response to adenovirus infection, revealing multiple potential pathways for further investigation.

Among these, *G0S2* has been implicated in apoptosis regulation, though its precise role remains context-dependent acting as either a pro- or anti-apoptotic factor depending on cellular conditions (ref. 28, 44 of the article). One intriguing hypothesis is that the upregulation of *G0S2*, *NAMPT*, and *BCL2A1* may collectively inhibit apoptosis; adenoviruses triggering a premature NETosis, though this warrants further experimental validation and could form the basis of future studies. Notably, *G0S2* has additional described functions in lipid droplet regulation, suggesting potential additional mechanisms worthy of exploration.

We performed our transcriptomic analysis at 90 minutes post-infection, as neutrophils begin exhibiting signs of cell death within hours of adenovirus exposure. While we did not document this phenomenon, we observed neutrophil aggregation beginning approximately 2 hours after adenovirus incubation. That factor necessitated our early timepoint selection to ensure single-cell RNA sequencing of isolated

neutrophils. Moreover, Kobayashi *et al.*'s examination of Staphylococcus aureus induced transcriptional changes in neutrophils demonstrated detectable modulation as early as 1 hour post-phagocytosis with increase changes at later timepoints (2-3 hours) [12].

5) The cell death inhibitor studies lack focus and are difficult to interpret. The major effect is observed by the necroptosis inhibitor GSK872 and EDTA, but never linked to the other data in the paper. What is the relationship between NETs and RIPK3? NETs and Ca+2, why was there no cell death by NETosis? Additional studies should be performed to determine if RIPK3 and Ca+2 inhibition also prevent neutrophil NET formation. What is the relationship of all of this to transcriptomic data?

In this study, we demonstrated that Ads induce NET formation and a RIPK1- and MLKL-independent, RIPK3-dependent lytic cell death. Previous research has shown a RIPK3-CamKII interaction in neuronal cells upon a flavivirus infection [13] supporting the hypothesis that CamKII may play a role in viral infection contexts, such as Ad-PMN interactions. Similarly, in cardiomyocytes, RIPK3 has been implicated in CamKII activation (ref. 61 of the manuscript). CamKII has been linked to the activation of the mitochondrial permeability transition pore (mPTP) and the production of mitochondrial ROS, both of which contribute to NETosis in PMNs (ref. 59 of the manuscript). Thus, RIPK3 activation may be mechanistically connected to NETosis.
Additionally, calcium signaling is known to promote NETosis by activating CamKII. Calcium can also activate calpain, which cleaves nesprin-1, weakening the nuclear envelope and facilitating NET release (ref. 58 of the manuscript). However, since not all pathways involved have been fully elucidated, we cautiously describe this process as a RIPK3- and calcium-dependent cell death accompanied by NET emission. To further validate that RIPK3 and calcium chelation decrease NET formation, quantitative assessment of NET formation would be necessary. We employed a NET quantification method, but the results under the control condition (PMA) were not fully convincing. Further optimization is needed, which could serve as a focus for future work**.** Regarding the transcriptomic data, we have discussed their implications and potential links to neutrophil death in the preceding sections.

6) There are off target effects for all the inhibitors listed and more specific inhibition approaches should be utilized to validate findings (eg. z-VAD blocks all caspases, apoptosis and pyroptosis, EDTA chelates all divalent cations and could have other effects on cellular membranes).

Only a pharmacological approach targeting PMNs is feasible, as these are terminally differentiated cells refractory to transfection, making a genetic approach complicated (including RNA interference). We therefore targeted several proteins in each signalling pathways to investigate all the main actors involved. Moreover, even though EDTA can chelate all bivalent cations and may impact cellular membranes, we confirmed that Ad phagocytosis still occurred in presence of 2.5 mM EDTA, as shown in S7 Fig. We can also measure that there was still some extracellular calcium in the medium with EDTA (see the answer to the point 11 of the reviewer 2). Concerning RIPK3, we performed new experiments with two other inhibitors (GSK840 and GSK843) to confirm the results with GSK 840. These new data are presented Fig. 7E and S13 Fig.

7) The re-infection/transduction study performed is artificial and lacks relevance. In an in vivo infection, where NETosis does not occur in isolation, would adenoviruses released from dying?NET neutrophils still be infectious?

The aim of this experiment was to determine whether Ads were still capable of transducing cells after phagocytosis and thus assess the impact of phagocytosis (and therefore ROS production and lytic enzyme activity) on Ads. Our results indicate that, within our experimental system, a fraction of Ad particles remained intact and functional even after prolonged exposure to phagocytic conditions.

In an *in vivo* infection model, it is therefore plausible that not all Ad would be eliminated by polymorphonuclear neutrophils (PMNs) at the infection site. However, an *in vivo* study is out of the

scope of the present study.

Part III – Minor Issues: Editorial and Data Presentation Modifications
Please use this section for editorial suggestions as well as relatively minor modifications of existing data that would enhance clarity.

Reviewer #1:

Minor points:

1) Is it really accurate to say that Ad3 and Ad5 use different intracellular trafficking (line 95)? Not sure that is what ref. 18 concludes.

We modified the reference.

2) Probably need at least one paragraph break between lines 207-241.

We added a paragraph break.

3) Don't use the word ignore on line 373. You are not ignoring the mechanism, it just remains unknown at this time.

We changed this word.

4) The method for recovery of virus from PMNs is not clear. On line 578 it is not clear if the harvested samples are total cells+media or just cell pellets, which are then freeze thawed. Please clarify.

The samples harvested were cells plus supernatant. This is now stated in material and methods.

5) Minor note: "Lore" indicates that purified Ad aggregated unless they are suspended in buffer containing Mg and Ca cations. This may even be stated in the 2005 Cotter paper (ref 17). This group seems to use basic PBS, without supplementation with Ca2+ and Mg2+, which can reduce viral sample homogeneity and experimental reproducibility. This is not an issue in serum or regular tissue culture medium, which contain both divalent cations.

We used to purify and store Ads in a phosphate-buffered saline solution without magnesium and calcium cations. Purified viruses were systematically analyzed by dynamic light scattering to check the hydrodynamic diameter and polydispersity of the Ads. A hydrodynamic diameter around 110 nm and a polydispersity below 0.2 was routinely obtained ruling out the presence of aggregates.

Reviewer #2: see above

Reviewer #3: The graphs should show individual data points.

We did it for all the graphs.

References

1. Song Z, Huang G, Chiquetto Paracatu L, Grimes D, Gu J, Luke CJ, et al. NADPH oxidase controls pulmonary neutrophil infiltration in the response to fungal cell walls by limiting LTB4. Blood. 2020;135: 891–903. doi:10.1182/blood.2019003525
2. Jankowski A, Scott CC, Grinstein S. Determinants of the phagosomal pH in neutrophils. Journal of Biological Chemistry. 2002;277: 6059–6066. doi:10.1074/jbc.M110059200
3. Perussia B, Dayton ET, Lazarus R, Fanning V, Trinchieri G. Immune interferon induces the receptor for monomeric IgG1 on human monocytic and myeloid cells. J Exp Med. 1983;158: 1092–1113. doi:10.1084/jem.158.4.1092
4. Guyre PM, Campbell AS, Kniffin WD, Fanger MW. Monocytes and polymorphonuclear neutrophils of patients with streptococcal pharyngitis express increased numbers of type I IgG Fc receptors. Journal of Clinical Investigation. 1990;86: 1892–1896. doi:10.1172/JCI114921
5. Hsu AY, Huang Q, Liu F, Balasubramanian A, Luo HR. Neutrophil death-more than meets the eye. Exp Hematol. 2025;150: 104857. doi:10.1016/j.exphem.2025.104857
6. Roos D, van Buul JD, Tool AT, Matute JD, Marchal CM, Hayee B, et al. Two CGD Families with a Hypomorphic Mutation in the Activation Domain of p67phox. J Clin Cell Immunol. 2014;5.
7. Kenny EF, Herzig A, Krüger R, Muth A, Mondal S, Thompson PR, et al. Diverse stimuli engage different neutrophil extracellular trap pathways. Elife. 2017;6. doi:10.7554/eLife.24437
8. Amjadi MF, Avner BS, Greenlee-Wacker MC, Horswill AR, Nauseef WM. Neutrophil-derived extracellular vesicles modulate the phenotype of naïve human neutrophils. J Leukoc Biol. 2021;110: 917–925. doi:10.1002/JLB.3AB0520-339RR
9. Hancock JT, Jones OT. The inhibition by diphenyleneiodonium and its analogues of superoxide generation by macrophages. Biochemical Journal. 1987;242: 103–107. doi:10.1042/bj2420103
10. Tazzeo T, Worek F, Janssen L. The NADPH oxidase inhibitor diphenyleneiodonium is also a potent inhibitor of cholinesterases and the internal Ca^{2+} pump. Br J Pharmacol. 2009;158: 790–796. doi:10.1111/j.1476-5381.2009.00394.x
11. Beaumel S, Verbrugge L, Fieschi F, Stasia MJ. CRISPR-gene-engineered *CYBB* knock-out PLB-985 cells, a useful model to study functional impact of X-linked chronic granulomatous disease mutations: application to the G412E X91+-CGD mutation. Clin Exp Immunol. 2023;212: 156–165. doi:10.1093/cei/uxad028
12. Kobayashi SD, Braughton KR, Palazzolo-Ballance AM, Kennedy AD, Sampaio E, Kristosturyan E, et al. Rapid Neutrophil Destruction following Phagocytosis of Staphylococcus aureus. J Innate Immun. 2010;2: 560–575. doi:10.1159/000317134
13. Estevez I, Buckley BD, Lindman M, Panzera N, Chou T-W, McCourt M, et al. The kinase RIPK3 promotes neuronal survival by suppressing excitatory neurotransmission during central nervous system viral infection. Immunity. 2025;58: 666-682.e6. doi:10.1016/j.immuni.2025.01.017
